# The effects of lecanemab on cognitive function and hippocampal nerve regeneration in hypertensive AD mice

**DOI:** 10.1002/alz70861_108329

**Published:** 2025-12-23

**Authors:** Chuansheng Zhao, Xiuchun Zhang, Mengyuan Zhang, Cheng Lv

**Affiliations:** ^1^ The first hospital of China Medical University, Shenyang, Liaoning China

## Abstract

**Background:**

Alzheimer's disease (AD) is a neurodegenerative disorder of the central nervous system. Its hallmark pathological features include extracellular deposition of amyloid‐beta (Aβ) plaques and intracellular accumulation of neurofibrillary tangles formed by hyperphosphorylated tau (*p* ‐tau). Hypertension is an important comorbidity of AD. Lecanemab is a humanized monoclonal antibody, which can selectively neutralize and clear amyloid‐beta (Aβ) protein aggregates that cause AD neuropathy. At present, the efficacy of lecanemab in AD patients with hypertension remains uncertain. We explored the effects of lecanemab on cognitive function and hippocampal neurogenesis in hypertensive AD mice.

**Method:**

Research subjects included male 10‐month‐old APP/PS1 transgenic the AD mice and AngII mice, which were randomly divided into four groups: APP/PS1+PBS group, APP/PS1+Lecanemab group, APP/PS1+AngII+PBS group, and APP/PS1+AngII+Lecanemab group, with 6 mice in each group. Lecanemab (30 mg/kg) or PBS were administered intravenously via the tail vein once a week. After four weeks of continuous treatment, cognitive function was evaluated using the water maze test and the Y‐maze test. The deposition of amyloid‐beta (Aβ) protein in mouse brains was detected using immunofluorescence staining, while dendritic spine density and synaptic length were quantified through Golgi staining.

**Result:**

We found that Lecanemab intervention significantly improved cognitive performance in both APP/PS1 and APP/PS1+AngII mice. This was demonstrated in the Y‐maze test by an increased number of novel arm entries, longer exploration time, and greater exploration distance compared to the APP/PS1+PBS and APP/PS1+AngII+PBS groups. Lecanemab intervention can significantly enhanced cognitive performance in both APP/PS1 and APP/PS1+AngII mice in water mazes test, both the APP/PS1+Lecanemab and APP/PS1+AngII+Lecanemab groups showed a significant decrease in the time required to find the platform compared with their respective PBS‐treated control groups (APP/PS1+PBS and APP/PS1+AngII+PBS). Additionally, the APP/PS1+AngII+Lecanemab group showed a slightly shorter time to find the platform compared to the APP/PS1+Lecanemab group. Immunofluorescence staining revealed that Lecanemab intervention reduces hippocampal Aβ protein expression in APP/PS1 mice. Golgi staining demonstrated that Lecanemab significantly increased the density and length of hippocampal dendritic spines in mice, particularly in the APP/PS1+AngII+Lecanemab group.

**Conclusion:**

Lecanemab significantly ameliorated cognitive deficits in hypertensive AD model mice and significantly increased dendritic spine density and length.

